# Investigation of sex expression profiles and the cantharidin biosynthesis genes in two blister beetles

**DOI:** 10.1371/journal.pone.0290245

**Published:** 2023-08-18

**Authors:** Yuan-Ming Wu, Jia-Ran Li, Jiang Li, Tao Guo

**Affiliations:** 1 Department of Basic Medical College, Guizhou Medical University, Guiyang, Guizhou, The People’s Republic of China; 2 Department of Orthopedics, The Third Affiliated Hospital of Xinxiang Medical University, Xinxiang, Henan, The People’s Republic of China; 3 Department of Genomics-Center, Biozeron Shenzhen Incorporation, Shenzhen, Guangdong, The People’s Republic of China; 4 Department of Orthopedics, Guizhou Provincial People’s Hospital, Guiyang, Guizhou, The People’s Republic of China; University of Bari: Universita degli Studi di Bari Aldo Moro, ITALY

## Abstract

Cantharidin (CTD) is a well-established defensive toxin synthesized by blister beetles, displaying both therapeutic potential and toxicity. Among these beetles, *Hycleus cichorii* and *Hycleus phaleratus* are the two most commercially significant species due to their capacity to produce CTD in males. In this investigation, we conducted a gene expression profiling analysis of male and female individuals of these two species, utilizing the Illumina Hiseq4000 platform. We identified 7,983 expressed genes, including 2,823 differentially expressed genes (DEGs) shared by both male and female blister beetles. Nineteen genes related to CTD biosynthesis in the terpenoid backbone biosynthesis pathway were identified, including hydroxymethylglutaryl-CoA reductase (*HMGR*; EC:1.1.1.34), which demonstrated a significant correlation with CTD content. Furthermore, hydroxymethylglutaryl-CoA synthase (*HMGS*; EC:2.3.3.10) and isopentenyl-diphosphate Delta-isomerase (*IDI*; EC:5.3.3.2) were also found to be significantly up-regulated in males. Comparative analysis revealed that NADP+-dependent farnesol dehydrogenase (*FOHSDR*; EC:1.1.1.216) and farnesyl diphosphate synthase (*FDPS*; EC:2.5.1.1) had the highest copy number in these beetles, significantly higher than the copy number of the other four non-Meloidae insects. The analysis of the protein-protein interaction network of genes related to CTD biosynthesis revealed that the acetyl-CoA C-acetyltransferase (*ACAT*; EC:2.3.1.9) gene was the central gene, exhibiting greater expression in male blister beetles than in females. This study offers novel insights into the mechanisms of CTD biosynthesis in blister beetles and enhances our comprehensions of the association between particular genes and CTD content.

## Introduction

Cantharidin (C_10_H_12_O_4_, CTD), a vesicant synthesized by blister beetles belonging to the Meloidae family (Insecta: Coleoptera), is extensively employed as an anti-insect and antibacterial agent in agriculture and medicine globally [1–4]. Studies have indicated that CTD and its derivatives may have therapeutic potential for various types of cancer, including liver, lung, stomach, and esophageal cancers [5–8]. At the molecular level, the “terpenoid backbone biosynthesis” pathway is responsible for CTD synthesis in blister beetles [9]. Two pathways, namely the mevalonate (MVA) pathway and the methylerythritol 4-phosphate/deoxyxylulose 5-phosphate (MEP/DOXP) pathway, are involved in the synthesis of terpenoids in plants [10]. The biology, ecology, and medicinal applications of blister beetles have been extensively studied, as well as artificial breeding techniques [11–14]. Several articles have delved into the biosynthesis of CTD. Guan, Hao, et al. reported the draft genome of *Mylabris aulica* and identified 30 genes involved in the “terpenoid backbone biosynthesis” pathway, including two previously uncharacterized genes [15]. Another study suggested that methyl farnesoate epoxidase (*MFE*) and juvenile hormone epoxide hydrolase (*JHEH*) may be involved in the biosynthesis of CTD [16]. Additionally, qRT-PCR data showed that *MFE* and *JHEH* exhibit different expressional tendencies in both larval and adult blister beetles. Jiang, Lü et al. found that 3-hydroxy-3-methylglutary-CoA reductase (*HMGR*) and cytochrome P450 gene (*CYP4BM1*) also affect CTD production in *Epicauta chinensis* [17]. Furthermore, the findings also suggested that the fat body may play a more pivotal role in CTD biosynthesis in male *E*. *chinensis* after mating, and multiple tissues may be involved in the process.

CTD is a sesquiterpenoid biosynthesized primarily by adult male blister beetles and transferred to adult females as a defensive substance to safeguard their offspring [11, 12]. Moreover, CTD production in blister beetles varies at different development stages, and most blister beetles exhibiting sexual dimorphism with respect to CTD production [11, 13, 18, 19]. Male beetles produce significantly greater quantities of CTD within 5–30 days after emerging from their pupal stage, which increases with age [20]. In contrast, female beetles show low levels of CTD, which raises questions about the degree of dimorphism and whether there are any other genes that affect CTD synthesis in blister beetles.

RNA sequencing (RNA-seq) is a highly effective method for measuring gene expression levels and provides a strong foundation for future research. Gene networks play an important role in various organisms and systems, effectively helping to reveal the essential rules of a large number of biological processes and reactions in organisms [21]. The dry body of beetle has been a traditional medicine in China for the past 2000 years [22]. However, only *Hycleus cichorii* Linnaeus and *Hycleus phaleratus* Pallas are listed in the Pharmacopoeia of the People’s Republic of China and commonly used in artificial culture [20, 23–25]. Although four draft reference genomes of blister beetles have been published [15, 26, 27], only the gene sets of *H*. *cichorii* and *H*. *phaleratus* are currently available in public databases. This observation, along with the availability of correlational studies in these organisms [20, 23–25], prompted us to select *H*. *cichorii* and *H*. *phaleratus* as models for investigating CTD biosynthesis genes. We aimed to explore sex-based differences in gene expression levels between *H*. *cichorii* and *H*. *phaleratus* using the Illumina Hiseq4000 platform, by selecting samples from both females and males of *H*. *cichorii* and *H*. *phaleratus* obtained after 30 days of segregation breeding. In addition, we investigated CTD biosynthesis genes in the "terpenoid backbone biosynthesis" pathway and compared them with four non- Meloidae insects. We constructed an interaction network of CTD biosynthesis genes firstly. Our findings provide valuable insights into sex-related expression, as well as CTD biosynthesis in *Hycleus*.

## Materials and methods

### Sample collection and sequencing

Newly emerged *Hycleus cichorii* and *Hycleus phaleratus* adults were collected from soybean fields (N25°25′17.38″, E106°46′50.42″) in Luodian, Guizhou Province, China. After morphological species and gender identification, the insects were immediately stored in liquid nitrogen for RNA extraction. We sequenced female and male samples of these two beetles using the Illumina 4000 platform. Total RNA (~10 μg) was extracted from whole body using the TRIzol Reagent (Invitrogen, USA). Potential genomic DNA was removed by using DNase I. Poly (A) ^+^ RNA was extracted using poly-T oligo-coated magnetic beads. After purification, first-strand cDNA was synthesized using the Superscript II reverse transcriptase (Invitrogen, USA) and random hexamer primers. The cDNA was further converted into double-stranded DNA. The library was constructed using the TruSeq^®^ RNA Sample Prep Kit (Illumina). After library quality control, pair-end sequencing was performed using Illumina HiSeq^™^ 4000 platform at BGITech (Shenzhen, China).

### Data processing and mapping of clean reads to the reference gene

Raw reads were filtered by SOAPnuke [28] to obtain high-quality clean reads by removing reads with adaptor contamination, unknown nucleotides comprising more than 5%, and low-quality reads (>20% base with quality value less than 10 in a read). Subsequently, the clean reads were mapped to the reference gene [26] by Bowtie2 [29] program. In accordance with the recommendations of RSEM software (https://deweylab.github.io/RSEM/rsem-calculate-expression.html) and Zhao, X., et al [30], the bowtie parameters have been set to ’-q—phred64—sensitive—dpad 0—gbar 99999999—mp 1,1—np 1—score-min L,0,-0.1 -I 1 -X 1000—no-mixed—no-discordant -p 8 -k 200’.

### Gene expression and differential expressed genes analysis

Estimated mapped read count matrix was used to calculate fragments per kilobase per million mapped fragments (FPKM) by using RSEM [31] package. In order to reveal DEGs in female vs. male, DEGseq [32] analysis was performed at selection cutoff fold-change greater than or equal to 2 and *p*-value less than or equal to 0.001.

### Gene Ontology term enrichment analysis

To understand the functions of the DEGs, ClusterProfiler [33] was carried out. DEGs were significantly enriched in Gene Ontology (GO) terms when their *p*-value was less than 0.05.

### Identify the CTD-related genes and the interaction network of CTD-related genes

The key enzymes in the pathway of ‘terpenoid backbone biosynthesis’ (map00900) were annotated using the diamond program [34] with parameter of “—evalue 1e-05”. Four insect protein sequences, including *Tribolium castaneum* (TCAS), *Dendroctonus ponderosae* (DPON), *Bombyx mori* (BMOR), and *Drosophila melanogaster* (DMEL), were downloaded from Ensembl database (ftp://ftp.ensemblgenomes.org/pub/metazoa/release-49). Subsequently, the proteins were searched again the KEGG database with diamond program (—evalue 1e-05). The best hits were selected to obtain the CTD-related genes. Protein-protein interaction (PPI) network among the CTD-related genes was analyzed using the Search Tool for the Retrieval of Interacting Gene (STRING) [35] database, which included direct and indirect associations of proteins. The network figure was drawn by using Cytoscape software.

### qRT-PCR validation

To validate the expression of three key CTD-related genes between sexes, we conducted quantitative real-time PCR (qRT-PCR) analysis using the same source of insects described above. These insects were collected in Luodian, Guizhou, China. For each sample, we extracted total RNA from approximately 5 individuals and used the TransScript One-Step gDNA Removal and cDNA Synthesis SuperMix Kit (Transgen, China) to synthesize cDNA from 1 μg of total RNA following the manufacturer’s instructions. To normalize cDNA templates, we used UBE3A (ubiquitin-protein ligase E3A) and RPL22e (ribosomal protein) as internal references, as suggested by Vandesompele et al. [36]. We confirmed their expression stability following the method of Wang et al. [32]. The qRT-PCR was performed using the KAPA SYBR^®^FAST Universal qPCR Kit (KAPA BiOSYSTEMS, USA) on an optical 96-well plate with a Step One PlusTM Real Time PCR System (ABI, USA) in accordance with the manufacturer’s instructions. Thermal cycling conditions involved 40 cycles at 95°C for 5 s and 60°C for 30 s. After thermal cycling, we used the ABI Prism 7500 SDS software to analyze and adjust the results automatically. The data were exported to EXCEL for a 2-ΔΔCt analysis. We performed four independent biological replicates for each treatment.

## Results and discussion

### An overview of the RNA-Seq data

In the transcriptomic experiment, four cDNA libraries were prepared from samples of two adult blister beetles, one female and one male. The Illumina short read sequencing platform was utilized to obtain PE reads. Clean reads were generated from raw read sequences, yielded 30.8 Gb of paired-end clean data with the percent of Q20 was greater than 96% (Table 1). A total of 7,983 genes had an FPKM expression value above 1.0 in at least one sample (S1 Fig). The number of genes expressed in *H*. *cichorii* was more than that in *H*. *phaleratus*, and the number of genes expressed in female was more than that in male. The hierarchical clustering showed that the samples were generally divided by sex rather than breed (S2 Fig).

**Table 1:** The sequence quality of the two sister blister beetles.

	*H*. *cichorii*		*H*. *phalerata*	
	Female	Male	Female	Male
Total reads (Mb)	22.4	22.3	40.8	31.6
Total data (Gb)	4.5	4.5	12.3	9.5
GC content (%)	43.8	39.3	40.0	41.8
Clean Reads Q20 (%)	97.41	97.38	96.11	96.19

Note: Sequencing reads come from Wu, YM. et al. [45]

### Identification of DEGs between female and male

**Fig 1:**
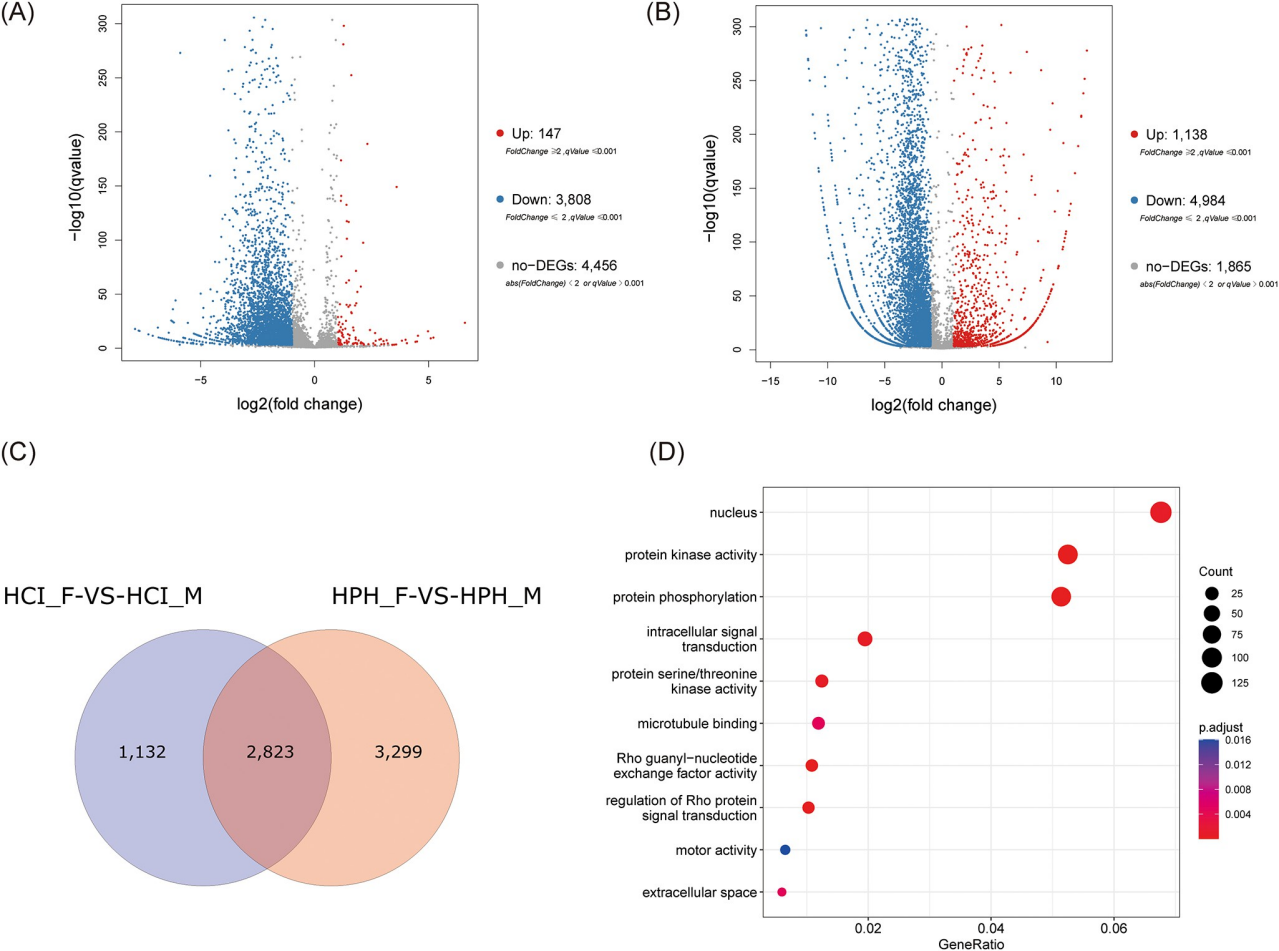
Results of the RNA-seq analyses. (A) Volcano plots in the female vs. male of *H*. *cichorii*. (B) Volcano plots in the female vs. male of *H*. *phaleratus*. The x-axis represents the log2 fold change in gene expression (fold change >2), and the y-axis represents the log10 of statistical significance. Green dots indicate down-regulated DEGs, while grey, red, and blue dots represent non-significant, up-regulated, and down-regulated genes, respectively. (C) Proportional Venn diagrams of DEGs. HCI_F refers to female *H*. *cichorii*, HCI_M refers to male *H*. *cichorii*, HPH_F refers to female *H*. *phaleratus*, and HPH_M refers to male *H*. *phaleratus*. (D) GO enrichment analysis of 2,823 DEGs. The top 10 GO terms are presented in a bar chart, ranked by gene count. The y-axis shows the names of the GO terms, while the shared number of terms is depicted as intersections of circles. Different colors represent different p.adjust values. The GeneRatio refers to the ratio of differentially expressed genes annotated in a particular GO term to all genes annotated in that term.

Differential analysis was performed to compare the level of gene expression between females and males. A total of 3,955 genes were identified as DEGs in *H*. *cichorii*, while 6,122 genes were identified as being DEGs in *H*. *phaleratus* (Fig 1A and 1B). Of the 3,955 DEGs in *H*. *cichorii*, 147 genes were up-regulated and 3,808 genes were down-regulated in the comparison of female vs. male. Similarly, of the 6,122 DEGs in *H*. *phaleratus*, 1,138 genes were up-regulated and 4,984 genes were down-regulated in the comparison of female vs. male. These findings suggest that in the adult stage of these two field blister beetles, females exhibit higher activity in gene expression than males. The gene expression levels of *CYP4BM1* and *MFE* in female *H*. *phaleratus* were found to be twofold higher than those in male *H*. *phaleratus*. There are three copies of *JHEH* gene in *H*. *cichorii*, of which two were significantly up-regulated in males. Accordingly, there are four copies of the *JHEH* gene in *H*. *phaleratus* and three of those copies were significantly up-regulated in males. Notably, 2,823 DEGs were shared in female vs. male (Fig 1C). GO terms were associated with the 2,823 DEGs to assess their putative biological roles. The most represented GO terms are protein kinase activity (97 DEGs), protein phosphorylation (95 DEGs), Rho guanyl-nucleotide exchange factor activity (20 DEGs), regulation of Rho protein signal transduction (19 DEGs), intracellular signal transduction (36 DEGs), protein serine/threonine kinase activity (23 DEGs), nucleus (125 DEGs), microtubule binding (22 DEGs), extracellular space (11 DEGs), motor activity (12 DEGs) (Fig 1D).

### Identification of genes involved in terpenoid backbone biosynthesis pathway

**Fig 2:**
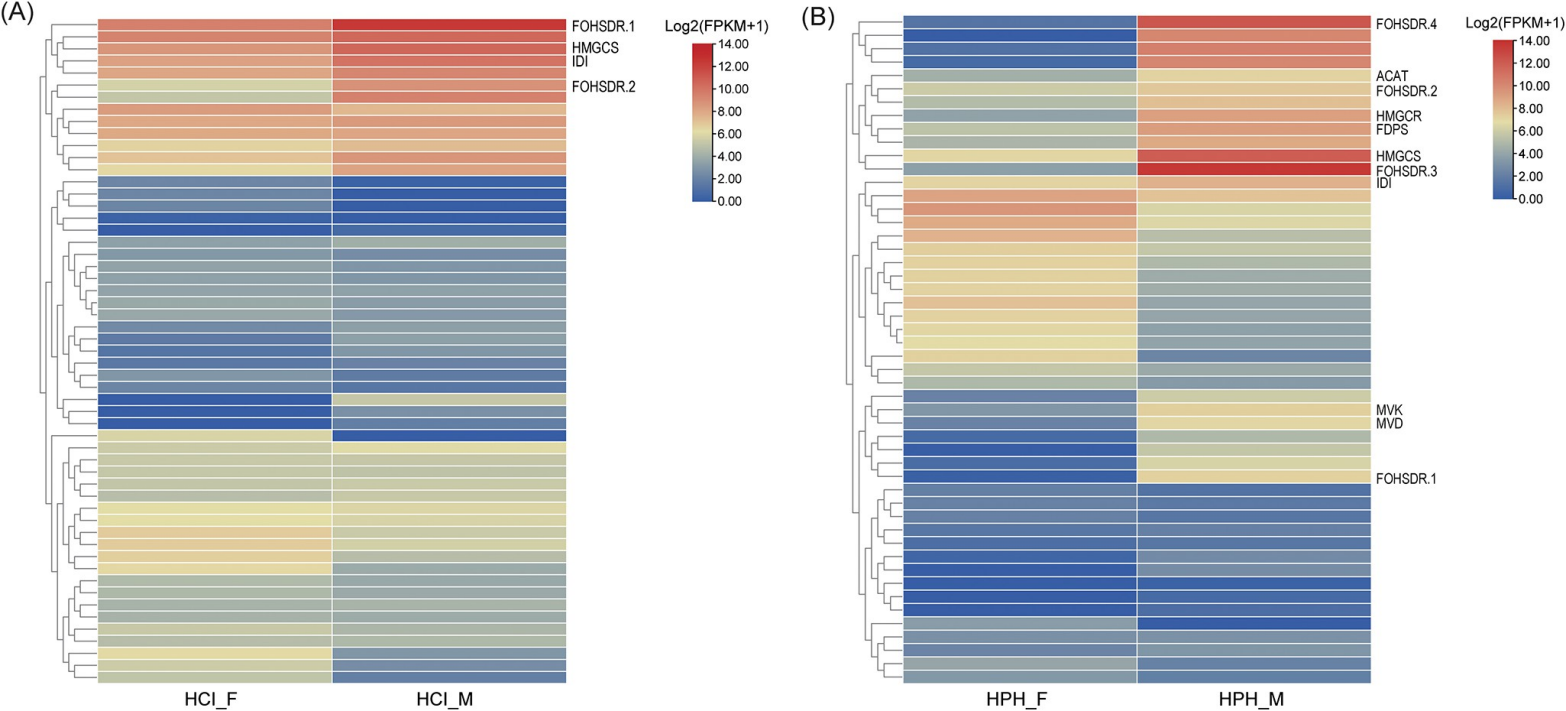
Heat map representation of CTD-related genes in *H*. *cichorii* and *H*. *phaleratus*. (A) Heat map of CTD-related genes in *H*. *cichorii*. (B) Heat map of CTD-related genes in *H*. *phaleratus*. Rows represent CTD-related genes, columns represent different samples. Bar in the upper right corner represents log 2 transformed FPKM values. Blue and red boxes represent genes showing lower and higher expression level, respectively. HCI_F refers to female *H*. *cichorii*, HCI_M refers to male *H*. *cichorii*, HPH_F refers to female *H*. *phaleratus*, and HPH_M refers to male *H*. *phaleratus*.

We examined the *Hycleus* genes involved in the “terpenoid backbone biosynthesis” pathway (map00900) to provide insights regarding CTD biosynthesis. In total, 19 CTD-related genes were detected, all of which are only involved in the MVA pathway (S3 Fig). Thirteen genes have a single copy, while 6 genes have multiple copies, yielded 56 genes in *H*. *cichorii* and 54 genes in *H*. *phaleratus* respectively (Table 2). The first two genes with the largest number of copies are *FOHSDR* and *FDPS*, respectively. Notably, the copy numbers of these two genes were significantly higher than those of four non-Meloidae insects, suggesting that these two genes may play a critical role in CTD production. We also utilized transcriptome data to study the gene expression pattern of these CTD biosynthesis genes. Among the 56 CTD genes in *H*. *cichorii*, 25 genes were up-regulated in males, four of which were DEGs, including *FOHSDR* (two copies), *HMGS*, and *IDI* (Fig 2A). Simultaneously, among the 54 CTD genes in *H*. *phaleratus*, 29 genes were up-regulated in males, eleven of which were DEGs, including *FOHSDR* (4 copies), *HMGR*, *ACAT*, *FDPS*, mevalonate kinase (*MVK*; EC:2.7.1.36), diphosphomevalonate decarboxylase (*MVD*; EC:4.1.1.33), *HMGS* and *IDI* (Fig 2B). Verification results from quantitative real-time PCR also revealed that *HMGS* and *IDI* were significantly up-regulated in males (S4 Fig).

**Table 2:** Number of CTD biosynthesis genes in six insects.

KO identifier	Symbol	Hcic	Hpha	Tcas	Dpon	Bmor	Dmel
K15890	*FOHSDR*	25	24	14	11	3	9
K00787	*FDPS*	6	5	2	1	2	1
K00021	*HMGR*	4	3	2	2	1	1
K00626	*ACAT*, *atoB*	3	3	2	4	1	3
K06013	*STE24*	3	4	3	2	3	5
K12505	*PDSS2*	2	2	1	2	5	1
K00587	*ICMT*, *STE14*	1	1	1	1	1	1
K00804	*GGPS1*	1	1	1	0	1	1
K00869	*MVK*, *mvaK1*	1	1	1	0	1	1
K01597	*MVD*	1	1	1	1	1	1
K01641	*HMGS*	1	1	1	1	1	1
K01823	*IDI*	1	1	1	1	1	1
K05954	*FNTB*	1	1	1	1	1	1
K05955	*FNTA*	1	1	1	1	1	1
K08658	*RCE1*, *FACE2*	1	1	1	1	1	1
K11778	*DHDDS*	1	1	1	1	2	1
K12504	*PDSS1*	1	1	2	3	3	2
K13273	*PMVK*	1	1	1	1	1	1
K19177	*NUS1*	1	1	1	1	1	1

Note: *Tribolium castaneum* (TCAS), *Dendroctonus ponderosae* (DPON), *Bombyx mori* (BMOR), *Drosophila melanogaster* (DMEL). FOHSDR: NADP+-dependent farnesol dehydrogenase; FDPS: farnesyl diphosphate synthase; HMGR: hydroxymethylglutaryl-CoA reductase; ACAT, atoB: acetyl-CoA C-acetyltransferase; STE24: STE24 endopeptidase; PDSS2: decaprenyl-diphosphate synthase subunit 2; ICMT, STE14: protein-S-isoprenylcysteine O-methyltransferase; GGPS1: geranylgeranyl diphosphate synthase, type III; MVK, mvaK1: mevalonate kinase; MVD: diphosphomevalonate decarboxylase; HMGS: hydroxymethylglutaryl-CoA synthase; IDI: isopentenyl-diphosphate Delta-isomerase; FNTB: protein farnesyltransferase subunit beta; FNTA: protein farnesyltransferase/geranylgeranyltransferase type-1 subunit alpha; RCE1, FACE2: prenyl protein peptidase; DHDDS: ditrans,polycis-polyprenyl diphosphate synthase; PDSS1: decaprenyl-diphosphate synthase subunit 1; PMVK: phosphomevalonate kinase; NUS1: dehydrodolichyl diphosphate syntase complex subunit NUS1. The genes of TCAS, DPON, BMOR and DMEL come from Ensembl [46].

### Interaction network of CTD biosynthesis-related genes

**Fig 3:**
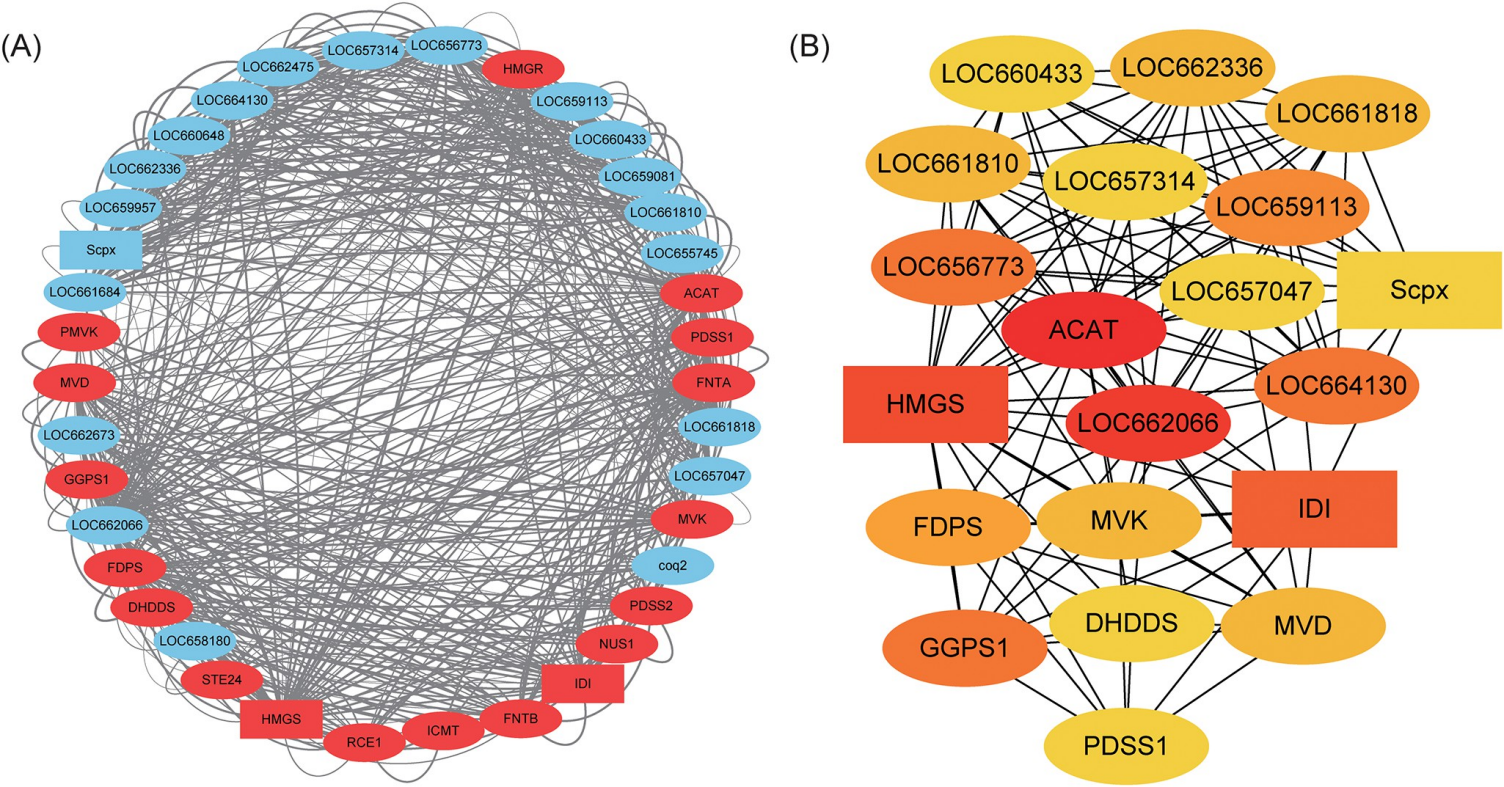
Interaction network of CTD-related genes. (A) Protein-Protein Interaction (PPI) network of all CTD-related genes. Red and blue ovals in nodes represent genes involved and not involved, respectively, in the “terpenoid backbone biosynthesis” pathway (map00900). The lines represent the regulation of relationship between two nodes. The darker the line color, the higher the interaction score. (B) Determination the top-ranked hub genes using the degree algorithm. The interaction network of top 20 nodes ranked by degree algorithm is shown. The more forward ranking is represented by a redder color.

To better understand the behavior of the genes and their interactions, we performed a PPI network analysis by using STRING database with CTD biosynthesis-related genes. In total, 20 non CTD biosynthesis-related genes interacted with 18 CTD biosynthesis-related genes (Fig 3A). The interaction network comprised 584 pairs of interactions, with an average of 15 pairs per gene. These genes were significantly enriched in 17 pathways, including “terpenoid backbone biosynthesis”, “valine, leucine and isoleucine degradation”, “metabolic pathways”, “butanoate metabolism”, “fatty acid degradation”, “fatty acid metabolism” and so on (S1 Table). The large number of interaction networks and numerous significantly enriched pathways indicated high functional diversity among CTD-related genes. To further investigate the core genes, we conducted a cytoHubba [37] analysis with degree algorithm to obtain the hub genes. The top 10 genes with the highest degree value were *ACAT* (also named *atoB*), *LOC662066*, *HMGS*, *IDI*, *LOC664130*, *LOC656773*, geranylgeranyl diphosphate synthase, type III (*GGPS1*), *LOC659113* and *FDPS* (Fig 3B). *ACAT* (also named *atoB*) was determined to be the core gene of the interaction network. Both RNAseq and Quantitative Real-Time PCR demonstrated that the expression level of *ACAT* (also named *atoB*) in males was higher than that in females (S4 Fig). The interaction network and key genes shed new light on our understanding of CTD biosynthesis.

### Discussion

CTD is a secondary metabolite derived from blister beetles and CTD biosynthesis is becoming a research hotspot. As reported, CTD has the potential to be a major compound used in resistance management [38]. More importantly, CTD was approved to be introduced by the USA Food and Drug Administration since 1997 and have been used to treat several cancers [5–7, 39]. *H*. *cichorii* and *H*. *phaleratus* are the most dominant species found China and are used in Chinese traditional medicine with a long period (Pharmacopoeia of the People’s Republic of China, 2005). Most blister beetles are sexually dimorphic with respect to CTD production, requiring a deeper understanding of the sex expression and CTD biosynthesis. Many studies have employed Illumina sequencing platform as it provides high-quality sequences for gene expression analysis. In this research, we identified 3,955 DEGs in *H*. *cichorii* and 6,122 DEGs in *H*. *phaleratus* with RNA-seq reads from both females and males of *H*. *cichorii* and *H*. *phaleratus* obtained after 30 days of segregation breeding. As breeding conditions mature, biological replicates should be included in the future study because they can improve the quality and reliability of analysis results [40]. The GO enrichment analysis of 2,823 DEGs shared in female vs. male showed that none of the significant GO terms related to CTD biosynthesis were included, suggesting that we should investigate the genes related to CTD biosynthesis. In total, 19 genes belonging to the “terpenoid backbone biosynthesis” pathway were obtained. Similar to in *M*. *cichorii*, these genes are only involved in MVA pathways. *FOHSDR* is the gene with the highest number of copies, and it has significantly more copies in the two blister beetles than in the other four non- Meloidae insects. The recent comparative genomic analysis of *M*. *aulica* suggested that none of the known genes related to CTD biosynthesis were included in expanded significantly gene families under GO and KEGG enrichment analyses [15]. Further studies are needed to determine whether the *FOHSDR* gene family is expanded in two blister beetles. Two previously uncharacterized CTD related genes were found in *M*. *aulica* with a draft genome [15]. We believe that with the improvement of genome assembly, the research of CTD-related genes will become more detailed, which is also one of the directions of our future efforts. *HMGR* was reported to exhibit a positive correlation in the fat body of male *E*. *chinensis* [17], and we found that the expression level of *HMGR* is higher in male *H*. *phaleratus* than in female *H*. *phaleratus*. Besides, two CTD-related genes, namely *HMGS* and *IDI*, have caught our attention because they were significantly up-regulated in male of two blister beetles. These findings were also confirmed by PCR experiments. Interaction networks provide insights for inferring the function of genes or proteins [41]. We found 584 pairs of interactions between CTD-related genes and non-CTD-related genes. The hub gene is often regarded as the key gene in gene regulatory network, which is of great significance to study the function and regulatory mechanism of this network [42, 43]. *ACAT* (also named *atoB*), the first enzyme that catalyzes the conversion of acetyl-CoA into acetoacetyl-CoA in the MVA pathway [44], was found to be at the core of CTD-related interaction network. Notably, *ACAT* also displayed higher expression in males compared to females. This is the first study to characterize the CTD-related interaction network with STRING database [35]. The diversity of interaction network gene pathway enrichment results indicated the functional diversity of CTD biosynthesis-related genes. The “insect hormone biosynthesis” pathway (map00981) maybe be one direction to study the synthesis of CTD in the future, especially cytochrome P450 family 15, subfamily A, polypeptide 1 (*CYP15A1*) and JH esterase (*JHE*) [9].

## Conclusions

In summary, sex expression profiles of *H*. *cichorii* and *H*. *phaleratus* were investigated by using Illumina deep-sequencing technology, and CTD biosynthesis-related genes were explored. A total of 2,823 DEGs were identified by comparing female and male individuals of the two blister beetle species. Additionally, nineteen CTD biosynthesis-related genes involved in the ‘terpenoid backbone biosynthesis’ pathway (map00900) were obtained. *HMGS* and *IDI*, the two key genes in the main MVA pathway, were up-regulated in male beetles from both species. Comparative analysis revealed that *FOHSDR* and *FDPS* are the top two highest copy number genes, and the copy number of these two genes is much higher than that of the other four non- Meloidae insects. A CTD biosynthesis-related gene network was firstly constructed using the STRING database, which revealed 584 pairs of interactions. *ACAT*, the core gene in the network, was up-regulated in male beetles. These results provide a broader view of CTD biosynthesis mechanism as well as provide valuable insights for genomics study in *Hycleus*.

## Supporting information

S1 Fig Statistic of expressed genes. HCI_F refers to female *H*. *cichorii*, HCI_M refers to male *H*. *cichorii*, HPH_F refers to female *H*. *phaleratus*, and HPH_M refers to male *H*. *phaleratus*.
(DOCX)

S2 Fig Hierarchical clustering between two blister beetles. HCI_F refers to female *H*. *cichorii*, HCI_M refers to male *H*. *cichorii*, HPH_F refers to female *H*. *phaleratus*, and HPH_M refers to male *H*. *phaleratus*.
(DOCX)

S3 Fig The “terpenoid backbone biosynthesis” KEGG pathway map. The highlighted red boxes represent the genes that are present in the *Hycleus*.
(DOCX)

S4 Fig qRT-PCR validation of atoB, *HMGS* and *IDI*. The relative quantities indicate the levels of putative transcripts normalized to the internal standard UBE3A (ubiquitin-protein ligase E3A) and RPL22e (ribosomal protein). The bars indicate the standard deviation of four repeats. Note: female of *H*. *cichorii* (HC-F), male of *H*. *cichorii* (HC-M), female of *H*. *phaleratus* (HP-F), male of *H*. *phaleratus* (HP-M).
(DOCX)

S1 Table The KEGG enrichment of CTD-related genes. (XLS)
