# Supplementary material for: Investigation of sex expression profiles and the cantharidin biosynthesis genes in two blister beetles
Source: PLoS One. 2023 Aug 18;18(8):e0290245. doi: 10.1371/journal.pone.0290245 (PMC10437994; doi:10.1371/journal.pone.0290245)
Supplement: S1 Fig — HCI_F refers to female H. cichorii, HCI_M refers to male H. cichorii, HPH_F refers to female H. phaleratus, and HPH_M refers to male H. phaleratus. (DOCX) [file pone.0290245.s001.docx]

**
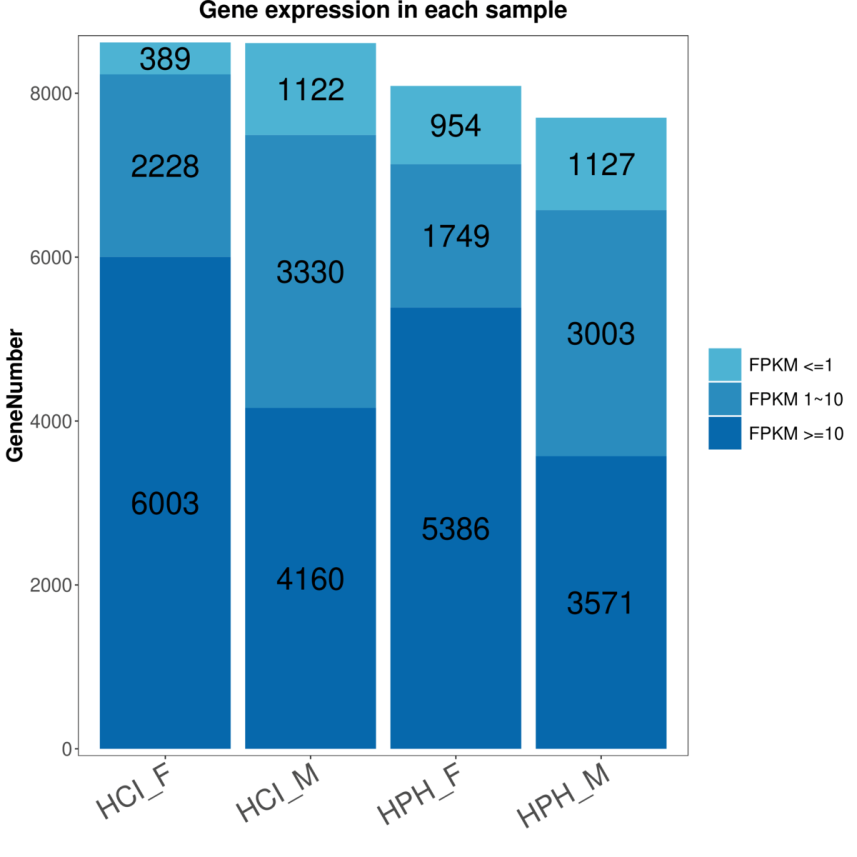
**

**S1 Fig. Statistic of expressed genes.** Note: female of *H. cichorii* (HCI_F), male of *H. cichorii* (HCI_M), female of *H. phaleratus* (HPH_F), male of *H. phaleratus* (HPH_M).
