# Supplementary material for: Investigation of sex expression profiles and the cantharidin biosynthesis genes in two blister beetles
Source: PLoS One. 2023 Aug 18;18(8):e0290245. doi: 10.1371/journal.pone.0290245 (PMC10437994; doi:10.1371/journal.pone.0290245)
Supplement: S3 Fig — The highlighted red boxes represent the genes that are present in the Hycleus. (DOCX) [file pone.0290245.s003.docx]

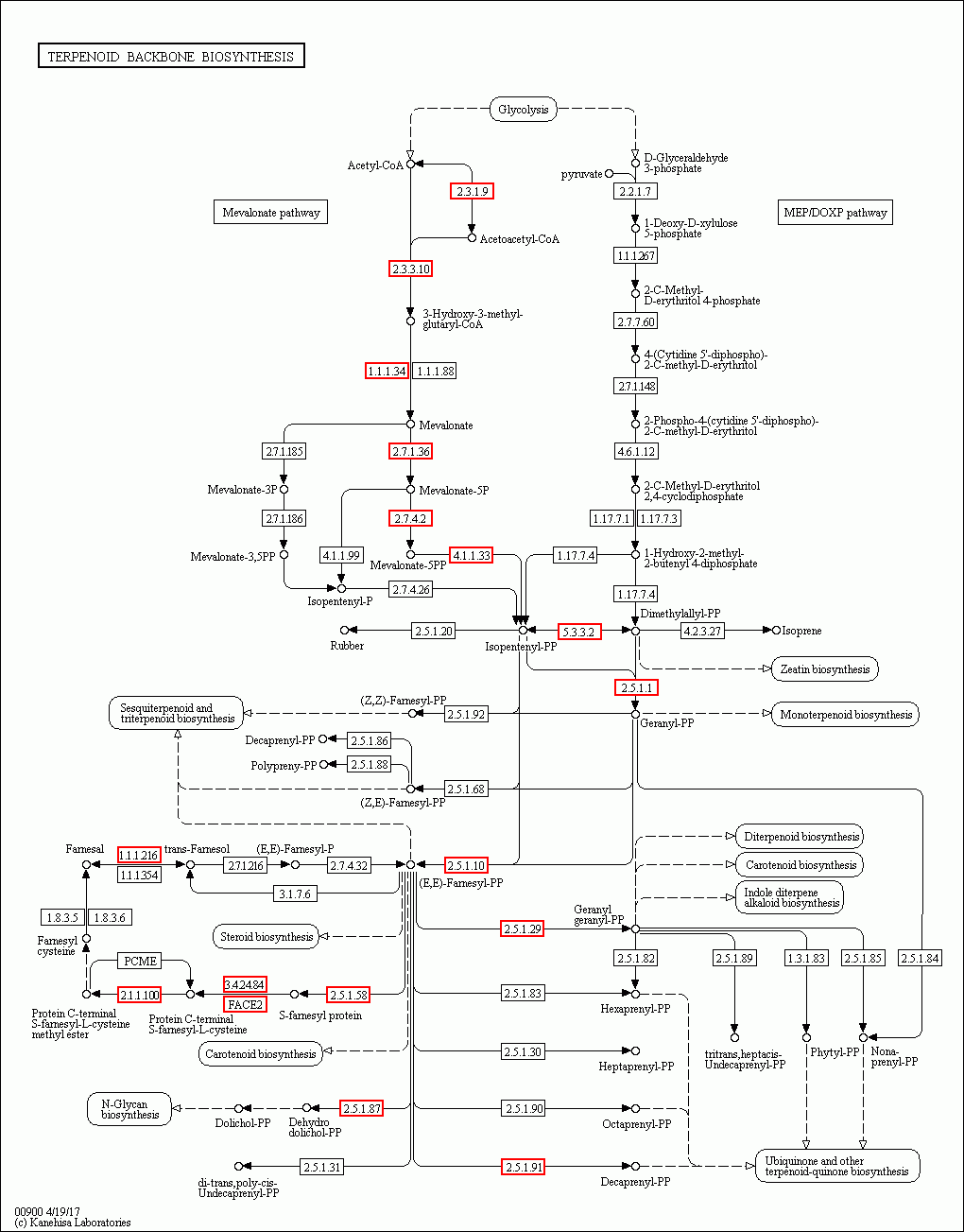
 **S3 Fig.** **The “Terpenoid backbone biosynthesis” KEGG pathway map**. The highlighted red boxes represent the genes that can be found in the *Hycleus*.
