# Supplementary material for: Investigation of sex expression profiles and the cantharidin biosynthesis genes in two blister beetles
Source: PLoS One. 2023 Aug 18;18(8):e0290245. doi: 10.1371/journal.pone.0290245 (PMC10437994; doi:10.1371/journal.pone.0290245)
Supplement: S4 Fig — The relative quantities indicate the levels of putative transcripts normalized to the internal standard UBE3A (ubiquitin-protein ligase E3A) and RPL22e (ribosomal protein). The bars indicate the standard deviation of four repeats. Note: female of H. cichorii (HC-F), male of H. cichorii (HC-M), female of H. phaleratus (HP-F), male of H. phaleratus (HP-M). (DOCX) [file pone.0290245.s004.docx]

**
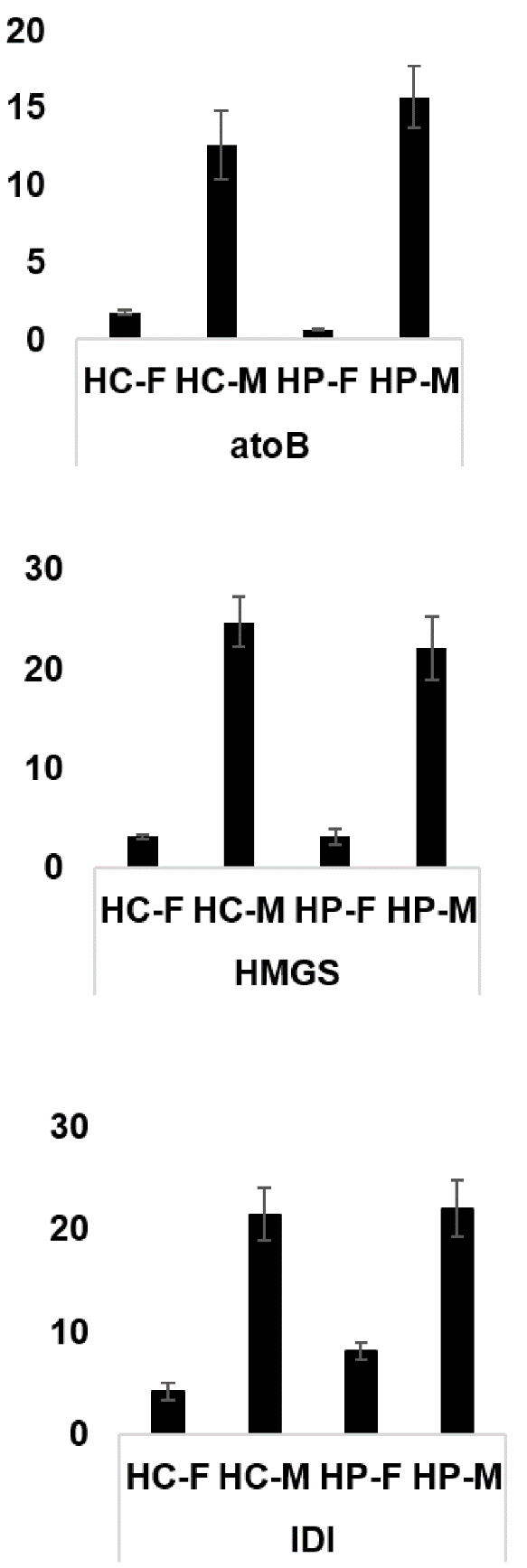
 S4 Fig. qRT-PCR validation of atoB, *HMGS* and *IDI*.** The relative quantities indicate the levels of putative transcripts normalized to the internal standard UBE3A (ubiquitin-protein ligase E3A) and RPL22e (ribosomal protein). The bars indicate the standard deviation of four repeats. Note: female of *H. cichorii* (HC-F), male of *H. cichorii* (HC-M), female of *H. phaleratus* (HP-F), male of *H. phaleratus* (HP-M).
